# Amyloid Fibrils from Hemoglobin

**DOI:** 10.3390/biom7020037

**Published:** 2017-04-11

**Authors:** Nadishka Jayawardena, Manmeet Kaur, Smitha Nair, Jenny Malmstrom, David Goldstone, Leonardo Negron, Juliet A. Gerrard, Laura J. Domigan

**Affiliations:** 1 School of Biological Sciences, The University of Auckland, Auckland 1010, New Zealand; gjay797@aucklanduni.ac.nz (N.J.); s.tipparaju@auckland.ac.nz (S.N.); d.goldstone@auckland.ac.nz (D.G.); j.gerrard@auckland.ac.nz (J.A.G.); 2 School of Biological Sciences, University of Canterbury, Christchurch 8041, New Zealand; manmeet.kaur@hi-aspect.com; 3 Department of Chemical and Materials Engineering, The University of Auckland, Auckland 1010, New Zealand; j.malmstrom@auckland.ac.nz; 4 The MacDiarmid Institute for Advanced Materials and Nanotechnology, Wellington 6140, New Zealand; 5 Callaghan Innovation, Lower Hutt 2010, New Zealand; leonardo.negron@callaghaninnovation.govt.nz; 6 School of Chemical Sciences, The University of Auckland, Auckland 1010, New Zealand

**Keywords:** amyloid fibrils, hemoglobin, nanofiber, nanofibril

## Abstract

Amyloid fibrils are a class of insoluble protein nanofibers that are formed via the self-assembly of a wide range of peptides and proteins. They are increasingly exploited for a broad range of applications in bionanotechnology, such as biosensing and drug delivery, as nanowires, hydrogels, and thin films. Amyloid fibrils have been prepared from many proteins, but there has been no definitive characterization of amyloid fibrils from hemoglobin to date. Here, nanofiber formation was carried out under denaturing conditions using solutions of apo-hemoglobin extracted from bovine waste blood. A characteristic amyloid fibril morphology was confirmed by transmission electron microscopy (TEM) and atomic force microscopy (AFM), with mean fibril dimensions of approximately 5 nm diameter and up to several microns in length. The thioflavin T assay confirmed the presence of β-sheet structures in apo-hemoglobin fibrils, and X-ray fiber diffraction showed the characteristic amyloid cross-β quaternary structure. Apo-hemoglobin nanofibers demonstrated high stability over a range of temperatures (−20 to 80 °C) and pHs (2–10), and were stable in the presence of organic solvents and trypsin, confirming their potential as nanomaterials with versatile applications. This study conclusively demonstrates the formation of amyloid fibrils from hemoglobin for the first time, and also introduces a cost-effective method for amyloid fibril manufacture using meat industry by-products.

## 1. Introduction

Amyloid fibrils are highly organized, insoluble, fibrillar aggregates that are formed from a wide range of soluble peptides and proteins [1]. Amyloid fibril formation in vitro can be triggered by destabilizing the native state of the protein by temperature and pH changes, chemicals, mutations, and shear flow [2]. Amyloid fibrils form via nucleation-dependent polymerization, which involves the self-assembly of partially unfolded intermediates to form the final structure [3]. The self-assembly properties [1,4,5] and high stability [6,7] of amyloid fibrils suggest the potential to utilize them as bionanomaterials [8]. Previous research has demonstrated the functionalization of fibrils for various applications such as biosensors [9,10,11,12,13], nanowires [14,15,16], nanocomposites [17,18], thin films [19], nanoporous matrices [20], hydrogels [21], and aerogels [22]. Their stability over a broad-range of temperatures, pHs, solvents, and proteases, allows amyloid fibrils to be exploited for many applications [13,23].

While the self-assembly properties of amyloid fibril formation potentially facilitate commercial scale production, the majority of amyloid fibrils have been formed from high-cost, purified protein/peptide sources. This is a major constraint in translating laboratory scale conceptual applications to commercial products [19,20,21,24,25]. Low-cost, crude protein sources such as whey [25], soy [26], and crystallin proteins extracted from fish, bovine, and deer eye lenses, have been used for amyloid fibril formation and functionalization [12,27,28]. This study focuses on utilizing bovine blood as a low-cost protein source for amyloid fibril synthesis.

To date, the literature on amyloid fibril formation from hemoglobin remains inconclusive, with no convincing evidence of a fibril form of dimensions and cross-β quaternary structure characteristic of amyloid fibrils [29,30,31]. Although β-sheet rich protein assemblies from hemoglobin have been presented in the literature, the diameter of these structures falls within the micrometer scale and the quaternary structure of these assemblies has not been well characterized [30,31].

In this study, we used hemoglobin obtained from bovine blood to produce amyloid fibrils, fully characterizing the fibril structure and morphology. The thioflavin T (ThT) assay was used to characterize β-sheet composition, while X-ray fiber diffraction studies provide insight into the quaternary structure of amyloid fibrils, highlighting a specific cross-β fingerprint [32,33]. The morphology and dimensions of amyloid fibrils were characterized by atomic force microscopy (AFM) and transmission electron microscopy (TEM) [34,35,36]. In addition, the stability of these amyloid fibrils was assessed under a range of temperatures, pHs, organic solvents, and proteases, such as would be required in an industrial processing setting. The fibril formation process was also performed at 250 mL, to confirm the scalability of the process.

## 2. Results

### 2.1. Amyloid Fibril Formation from Waste Blood Hemoglobin

It was found that nanofibers could be formed from the aqueous apo-form (AHB) when subjected to a low pH, salt, and an elevated temperature (pH 2.8, 125 mM NaCl, followed by incubation for 24 h at 80 °C). Amyloid fibrils were also formed under the same conditions using hemoglobin extracted from bovine waste blood by red blood cell (RBC) lysis, which was then purified to homogeneity, as described in the Methods section (Figure S1 and S2).

### 2.2. Characterisation of AHB Nanofibers

#### 2.2.1. Thioflavin T Fluorescence

**Figure 1:**
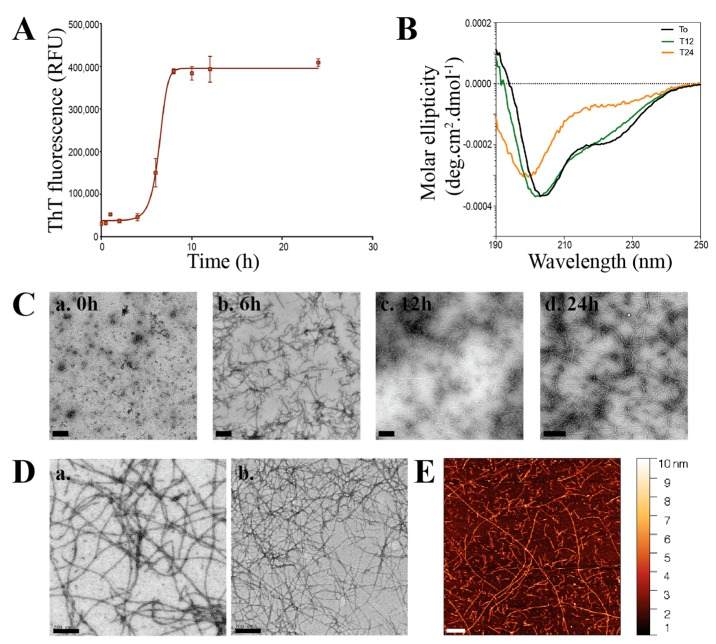
Formation of amyloid fibrils from hemoglobin. (**A**) Thioflavin T (ThT) fluorescence intensity overtime of aqueous apo-hemoglobin (AHB) solution extracted from pure hemoglobin after incubating at pH 2.8, 125 mM NaCl, 80 °C for 24 h. Each reading represents an average of triplicate well readings, with the error bars representing the standard error of mean. (**B**) Far ultraviolet (UV) circular dichroism (CD) spectrum of AHB solution following incubation at pH 2.8, 125 mM NaCl, 80 °C for 0 h (black), 12 h (green), 24 h (orange); (**C**) transmission electron microscopy (TEM) images of AHB solution following incubation at pH 2.8, 125 mM NaCl, 80 °C for 0 h (**a**), 6 h (**b**), 12 h (**c**), 24 h (**d**). Scale bars are 200 nm. (**D**) TEM images of AHB fibrils formed from pure hemoglobin (**a**) and waste blood hemoglobin (**b**). All the images were captured at 15,000 magnifications. Scale bars represent 200 nm. (**E**) Atomic force microscopy (AFM) image of AHB fibrils formed from waste blood hemoglobin. Images were acquired in air using tapping mode at a resonance frequency of 175 kHz and force constant of 1.5 N/m to 15 N/m. Scale bar represents 500 nm.

The ThT fluorescence of AHB under denaturing conditions shows a sigmoidal growth curve, characteristic of amyloid fibrils (Figure 1A). There is a lag phase of approximately 6 h, followed by an exponential growth phase, which then reaches a plateau. Protein aggregation in both pure AHB and that extracted from bovine blood was inferred by a cloud-like appearance of the solution after a 24-h incubation period. This was confirmed to be amyloid fibril formation by an increase in the extrinsic ThT fluorescence compared to that of the native protein (Figure S3).

#### 2.2.2. Circular Dichroism

Changes in the secondary structure of AHB during fibril formation were followed by far-ultraviolet (UV) circular dichroism (CD) (Figure 1B). By following the conformational changes over time (0 to 24 h), it is possible to view that there is a clear change in the secondary structure of AHB from α-helical to a β-sheet rich structure, characteristic of amyloid fibrils.

#### 2.2.3. TEM Imaging

TEM imaging of AHB under a denaturing condition shows the progression from oligomers to mature fibrils (Figure 1C, a–d) TEM imaging visually confirmed the presence of nanofibers in the treated AHB solutions obtained from both pure hemoglobin and that isolated from waste blood (Figure 1D).

#### 2.2.4. AFM Imaging and Analysis

**Figure 2:**
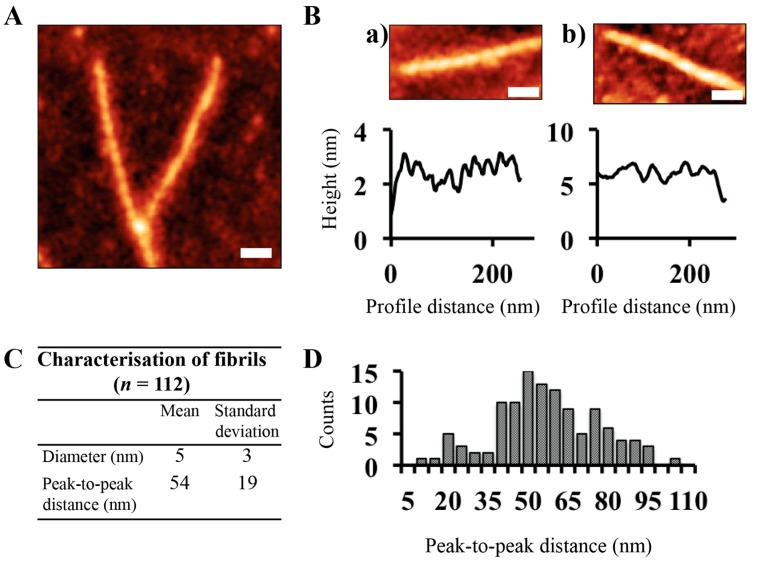
AFM analysis of hemoglobin amyloid fibrils. (**A**) A representative AFM micrograph of AHB fibrils formed from waste blood hemoglobin. Images were acquired in air using a tapping mode at a resonance frequency of 175 kHz and force constant of 1.5 N/m to 15 N/m. Scale bar is 50 nm. (**B**) Representative line traces along individual fibrils. Scale bar is 50 nm. (**C**) Table detailing the characterization of fibrils in terms of the diameter and peak-to-peak distances; (**D**) Distribution of peak-to-peak distances of hemoglobin amyloid fibrils (*n* = 112).

To perform statistical analysis, we measured the height profiles of approximately 100 fibrils. Because the feature width is dependent on the size and shape of the tip, height measurements were used to characterize fibril cross-sections. AHB nanofibers had mean diameters of 5 ± 3 nm. Atomic force microscopy images were also used to observe and measure the periodic height fluctuation along the fibril length. Figure 2B shows representative images and line traces of fibrils with two different peak-to-peak distances; 20 nm (Figure 2B, a) and 50 nm (Figure 2B, b), with the increased period leading to an increased fibril diameter. An analysis of the fibrils found the average peak-to-peak distance to be 54 nm (Figure 2C). Histogram analysis shows the presence of a variety of fibril periodicities (inferred by the peak-to-peak distance, Figure 2D).

#### 2.2.5. X-ray Fiber Diffraction

**Figure 3:**
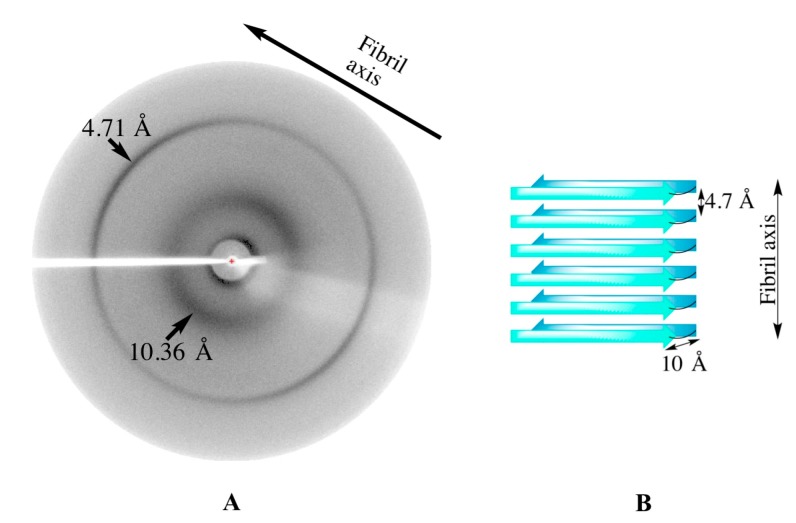
X-ray fiber diffraction pattern of AHB fibrils (**A**) and schematic diagram of the cross-β quaternary structure of amyloid fibrils (**B**). X-ray fiber diffraction showed characteristic reflections at 4.71 Å and 10.36 Å, corresponding to the molecular spacing between β-strands and β-sheets, respectively, as shown in (**B**). The X-ray fiber diffraction images were analyzed using Adxv software (version 1.9.12, Andrew Arvai, The Scripps Research Institute, La Jolla, CA, USA).

Nanofibers were also characterized by X-ray fiber diffraction, to confirm that the β-sheets were a constituent of the cross-β quaternary structure characteristic of amyloid fibrils (Figure 3B). Reflection arcs on the meridian and the equator accounted for distances of 4.71 Å and 10.36 Å, respectively.

### 2.3. Stability of AHB Fibrils

#### 2.3.1. Solvent Stability of AHB Fibrils

The stability of AHB fibrils was first evaluated in industrial solvents commonly encountered during microfabrication processes: methanol (MeOH), ethanol (EtOH), isopropanol (MeCH(OH)Me), dimethylsulfoxide (DMSO), and acetonitrile (MeCN). The maintenance of fibril integrity over a 24 h period was investigated by the ThT assay and TEM analysis.

**Figure 4:**
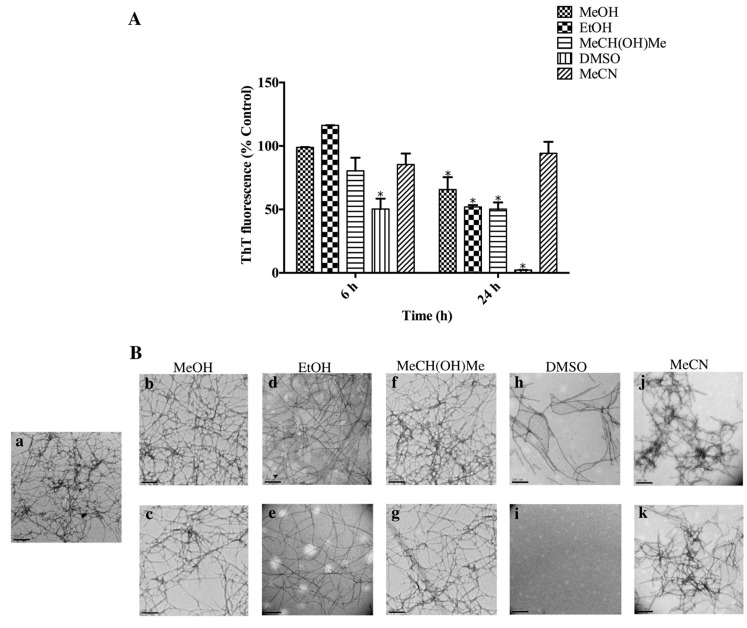
Solvent stability of AHB fibrils. (**A**) ThT fluorescence of AHB fibrils suspended in MeOH, EtOH, MeCH(OH)Me, dimethyl sulfoxide DMSO, and MeCN at 6 and 24 h, following buffer exchange to the appropriate solvent. ThT fluorescence is presented as % control (0 h). Each reading represents an average of triplicate well readings, with the error bars representing the standard error of mean. * Significantly different (two-way analysis of variance (ANOVA) followed by Bonferroni post hoc test) when compared to control, *p* < 0.05. (**B**) Representative TEM micrographs of AHB fibrils suspended in (**a**) fibrillation buffer; MeOH at (**b**) 6 h, (**c**) 24 h; EtOH at (**d**) 6 h, (**e**) 24 h; MeCH(OH)Me at (**f**) 6 h, (**g**) 24 h; DMSO at (**h**) 6 h, (**i**) 24 h; MeCN at (**j**) 6 h, (**k**) 24 h. The images were captured at 15,000× magnification and the scale bars represent 200 nm.

In the presence of MeOH, EtOH, and MeCH(OH)Me (100%), AHB fibrils showed no change in ThT fluorescence up to 6 h, followed by a decrease at the 24 h time point (Figure 4A). Despite a decrease in the ThT fluorescence at 24 h, TEM imaging showed there were still a large number of AHB fibrils present (Figure 4B), with no change in morphology. AHB fibrils incubated in DMSO were not stable, with significant fibril dissolution observed at the 6 h and 24 h time points, as demonstrated by the decrease in ThT fluorescence (Figure 4A) and TEM images (Figure 4B). MeCN did not induce AHB fibril dissociation over a period of 24 h, as indicated by the minimal loss of the ThT fluorescence, when compared to the 0 h control (Figure 4A). TEM imaging showed AHB fibrils to be present under these conditions (Figure 4B).

#### 2.3.2. Temperature Stability of AHB Fibrils

**Figure 5:**
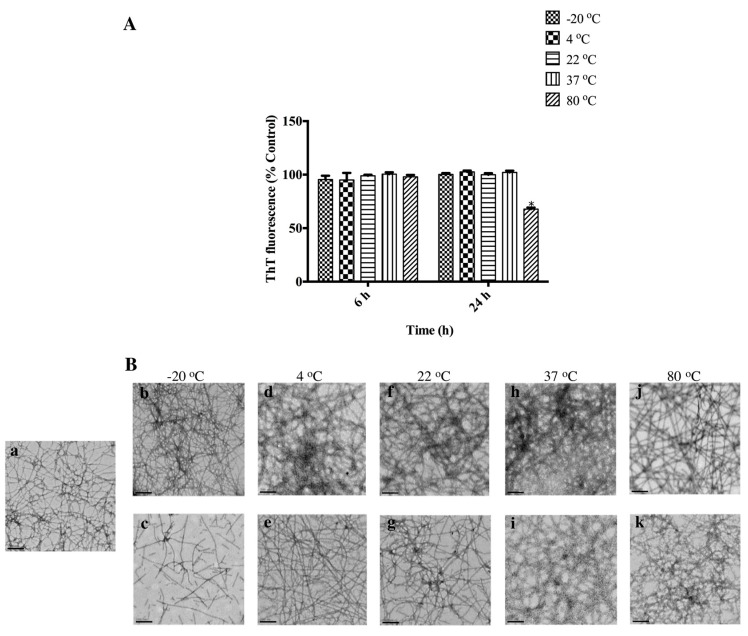
Temperature stability of AHB fibrils. (**A**) ThT fluorescence of AHB fibrils resuspended in the fibrillation buffer incubated at −20 °C, 4 °C, 22 °C, 37 °C, and 80 °C. ThT readings were reported post-buffer exchange. ThT fluorescence is presented as % control (0 h). Each reading represents an average of triplicate well readings, with the error bars representing the standard error of mean. * Significantly different (two-way ANOVA followed by Bonferroni post hoc test) when compared to control, *p* < 0.05. (**B**) Representative TEM micrographs of AHB fibrils suspended in (**a**) fibrillation buffer; −20 °C at (**b**) 6 h, (**c**) 24 h; 4 °C at (**d**) 6 h, (**e**) 24 h; 22 °C at (**f**) 6 h, (**g**) 24 h; 37 °C at (**h**) 6 h, (**i**) 24 h; 80 °C at (**j**) 6 h, (**k**) 24 h. The images were captured at 15,000× magnification and the scale bars represent 200 nm.

In this study, we investigated AHB fibril stability at −20 °C, 4 °C, 22 °C (room temperature), 37 °C, and 80 °C, following temperatures investigated in the literature by Kaur and co-workers [23]. AHB fibrils were stable at all temperatures, as suggested by the maintenance of ThT fluorescence (Figure 5A). The only exception was the decrease in ThT fluorescence when fibrils were heated at 80 °C for 24 h. Nonetheless, TEM micrographs of AHB fibrils incubated at 80 °C showed that fibrils were still present (Figure 5B). TEM micrographs of AHB fibrils stored at 37 °C, 22 °C, and 4 °C showed that AHB fibrils were stable at these temperatures, with no apparent changes in the morphology (Figure 5B). The storage of AHB fibrils at −20 °C caused a change in the fibril morphology for fragmented fibrils observed at the 6 h and 24 h time points (Figure 5B).

#### 2.3.3. AHB Fibril Stability under a Range of pHs

**Figure 6:**
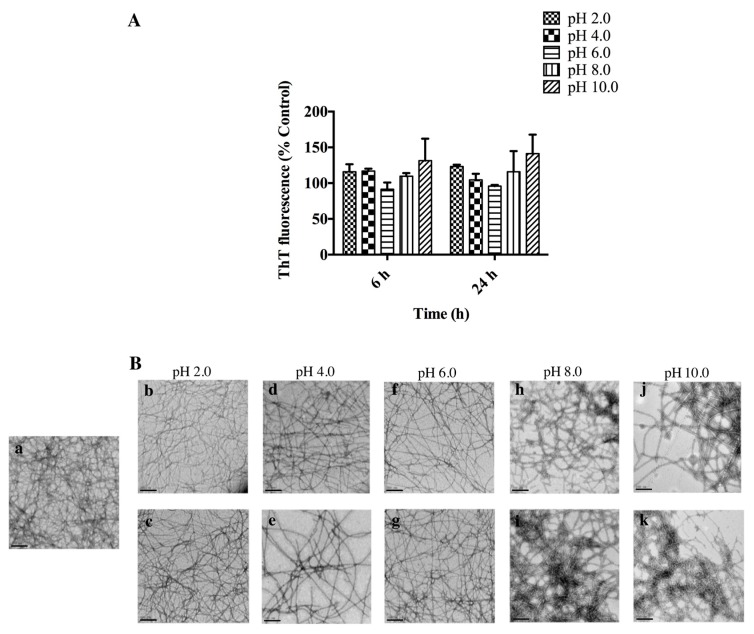
pH stability of AHB fibrils. (**A**) ThT fluorescence of AHB fibrils resuspended at pH 2.0 (30 mM glycine), 4.0 (30 mM sodium acetate), 6.0 (30 mM sodium citrate), 8.0 (30 mM Tris HCl), and 10.0 (60 mM sodium phosphate). ThT readings were reported post-buffer exchange with the original buffer used for fibrillation. ThT fluorescence is presented as % control (0h). Each reading represents an average of triplicate well readings, with the error bars representing the standard error of mean. * Significantly different (two-way ANOVA followed by Bonferroni post hoc test) when compared to control, *p* < 0.05. (**B**) Representative TEM micrographs of AHB fibrils suspended in (**a**) fibrillation buffer; pH 2.0 at (**b**) 6 h, (**c**) 24 h; pH 4.0 at (**d**) 6 h, (**e**) 24 h; pH 6.0 at (**f**) 6 h, (**g**) 24 h; pH 8.0 at (**h**) 6 h, (**i**) 24 h; pH 10.0 at (**j**) 6 h, (**k**) 24 h. The images were captured at 15,000× magnification and the scale bars represent 200 nm.

In order to determine the pH stability of AHB fibrils, fibrils were resuspended in appropriate buffers at pH 2.0, 4.0, 6.0, 8.0, and 10.0 [7]. The stability of fibrils was examined over a period of 24 h via the ThT assay and TEM imaging. AHB fibrils showed resistance towards both acidic and alkaline pHs, as well as common buffer components, which was demonstrated by stable ThT fluorescence throughout the experiment (Figure 6A). The presence of AHB fibrils was confirmed by TEM micrographs (Figure 6B).

#### 2.3.4. Protease Resistance of AHB Fibrils

**Figure 7:**
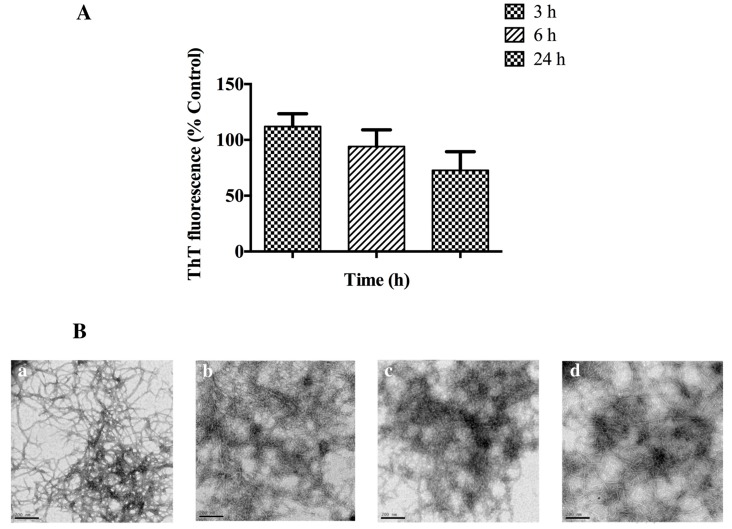
Protease resistance of AHB fibrils. (**A**) ThT fluorescence of AHB fibrils incubated with trypsin in 50 mM Tris HCl, 1 mM CaCl_2_ at pH 8.0 and 37 °C. ThT readings were reported post-buffer exchange with the original buffer used for fibrillation. ThT fluorescence is presented as % control (0 h). Each reading represents an average of triplicate well readings, with the error bars representing the standard error of mean. (**B**) Representative TEM micrographs of AHB fibrils treated with trypsin at (**b**) 3 h, (**c**) 6 h, and (**d**) 24 h time points with respect to (**a**) the control. The images were captured at 15,000× magnification and the scale bars represent 200 nm.

We assessed the stability of AHB fibrils in the presence of trypsin (see Experimental). The digestion of AHB fibrils was assessed up to the 24 h time point by the ThT assay and TEM imaging, and showed AHB fibrils to be resistant to trypsin digestion (Figure 7). Although the ThT fluorescence of the fibril samples treated with trypsin decreased with incubation time, there was no significant reduction in the extrinsic fluorescence at the 3 h and 6 h time points (Figure 7A). TEM micrographs visually confirmed the presence of AHB fibrils, although the network structure appeared to be altered, with a tighter network formed following trypsin treatment (Figure 7B). After 24 h incubation with trypsin, there appears to be some amorphous aggregate present, suggesting that some fibril digestion may be taking place.

### 2.4. Scaled up Formation of AHB Fibrils from Bovine Waste Blood

AHB fibrils could be formed in up to 250 mL volumes, with amyloid fibril formation confirmed by the ThT assay and TEM imaging (Supplementary Figure S4).

## 3. Discussion

### 3.1. Amyloid Fibril Formation from Waste Blood Hemoglobin

The literature reports some studies on hemoglobin fibril formation, but there is no definitive evidence that these fibrils are of an amyloid nature, or have the characteristic stability of amyloid fibrils [30,31]. In this study, commercially available pure hemoglobin, and that extracted from bovine blood, was shown to form amyloid fibrils, following the conversion to its apo-form (see liquid chromatography mass spectrometry (LC–MS) in Figure S2) in order to remove contaminating iron species from the final nanofiber solution.

### 3.2. Characterisation of AHB Nanofibers

The ThT assay, CD, TEM imaging, and X-ray fiber diffraction confirmed amyloid fibril formation from apo-hemoglobin extracted from bovine waste blood. The X-ray fiber diffraction of AHB resulted in the characteristic X-ray fingerprint of amyloid fibrils (Figure 3A). Reflection arcs on the meridian and the equator accounted for distances of 4.71 and 10.36 Å, which represent the molecular spacing between β-strands and β-sheets, respectively [32].

Detailed AFM analysis showed the fiber morphology to be left-handed twisted ribbons (Figure 2A). Contrary to previous studies on hemoglobin fibrils [30,31], AHB nanofibers had mean diameters of 5 ± 3 nm, characteristic of amyloid fibrils [34,35,36] (Figure 2C). Similar to what has been observed for β-lactoglobulin fibrils [37], we observed the presence of a periodic structure in hemoglobin fibrils with different periods. Histogram analysis shows the presence of a variety of fibril periodicities, representative of the hierarchical assembly of hemoglobin from protofilaments to mature amyloid fibrils.

### 3.3. Stability of AHB Fibrils

#### 3.3.1. Solvent Stability of AHB Fibrils

The stability of amyloid fibrils is important when considering their applications as nanomaterials [7]. The processing of amyloid fibrils for numerous applications such as biosensors, nanowires, and hydrogels often requires extreme temperatures, pHs, salinity, and solvents [11,12,14,15,19,38,39]. The stability of AHB fibrils was first evaluated in industrial solvents commonly encountered during microfabrication processes for nanowire and biosensor manufacturing [10,12,15,40]. Previous studies have shown that some amyloid fibrils undergo changes in morphology in the presence of certain solvents, which may affect their mechanical properties [41]. For example, 50% MeCN solutions have previously been reported to cause the formation of circular fibrillar assemblies [40]. Most of these microfabrication processes require short processing conditions (<6 h), although we have carried out solvent incubation for up to 24 h.

In the presence of MeOH, EtOH, and MeCH(OH)Me (100%), AHB fibrils showed no change in ThT fluorescence up to 6 h, followed by a decrease at the 24 h time point (Figure 4A). These results were in agreement with the previous studies, which have demonstrated the stability of whey, insulin, and crystallin fibrils in MeOH, EtOH, and MeCH(OH)Me [10,23]. As previously observed, the decrease in ThT fluorescence at 24 h could be linked to the degradation or solubilisation of intact AHB fibrils [41]. TEM confirmed no change in fibril morphology under these conditions. The stability of AHB fibrils in the tested alcohols may be related to the high non-polarity of these alcohols, which has been speculated to strengthen the hydrogen bond network in the fibrils [42].

AHB fibrils were not stable in DMSO, in agreement with the literature on fibrils derived from other protein sources [23]. The dissolution of AHB fibrils by DMSO is consistent with the disruption of the hydrogen bond network, which stabilizes the fibril structure [42]. AHB fibrils were stable in MeCN, another polar aprotic solvent, while other studies in the literature have demonstrated amyloid fibril solubility in MeCN [23,28,43].

#### 3.3.2. Temperature Stability of AHB Fibrils

Amyloid fibrils exhibit a remarkable thermal stability at temperatures as high as 284 °C [44], which may facilitate their use in a variety of platforms where high temperatures are encountered for sterilization or fabrication [13,40]. In contrast, freezing has been shown to fragment amyloid fibrils, as seen with insulin fibrils subjected to repeated freeze-thaw cycles [15], a mechanism that may be exploited for the production of fibril seeds.

In this study, temperatures were chosen as representative of those encountered in an industrial setting for various nanotechnology applications and storage purposes [13,23]. In agreement with previous studies conducted by Kaur and co-workers [23], AHB fibrils were stable at all temperatures, as suggested by the maintenance of ThT fluorescence (Figure 5A). The stability of fibrils at 22 °C and 4 °C is desirable from a storage point of view, to preserve fibril samples at readily accessible temperatures, and therefore, stability is required at these temperatures for prolonged periods of time. Some of the applications of amyloid fibrils, such as cell adhesion, require the incubation of fibrils and cells at 37 °C [45]. Following storage at −20 °C, there was a change in fibril morphology, with fibrils becoming fragmented. This was not unexpected, since previous studies have shown the fragmentation of insulin fibrils stressed with repeated freeze-thaw cycles. The significance of fibril fragmentation has been described in the literature in terms of its increased cytotoxicity when compared to long counterparts and its ability to seed the growth of new fibrils [46,47]. Therefore, the frozen conditions may not be an ideal mean of shipping AHB fibril samples due to their susceptibility to fragmentation, and alternative temperatures (4 °C) should be considered in such situations.

#### 3.3.3. AHB Fibril Stability under a Range of pHs

Another desirable feature for a nanocomponent is the ability to survive acidic and alkaline conditions [13,48]. Even though the role of pH on amyloid fibril formation has been well documented, limited studies have investigated the pH stability of amyloid fibrils post-formation [23,25,49]. Among the fibrils investigated for their pH stability, crystallin nanofibers have shown a superior stability at a pH range of 2.0–11.0 [23]. In contrast, whey and insulin nanofibers showed a higher resistance towards the acidic pHs in which they were formed and less stability towards alkaline pHs [7,23]. AHB fibrils showed resistance towards both acidic and alkaline pHs, as well as common buffer components. It was evident that, unlike whey and insulin fibrils [23], AHB fibrils did not dissociate or structurally rearrange under alkaline conditions. The observed pH resistance of AHB fibrils suggested the absence of any significant disruption of electrostatic interactions in the fibrillar structure, maintaining the fibril integrity and morphology under pH values away from the fibrillation pH, as observed in other systems [7].

#### 3.3.4. Protease Resistance of AHB Fibrils

A useful characteristic conferred by the highly stable cross-β quaternary structure of amyloid fibrils is their resistance towards a wide range of proteases [7,50]. Most of the proteolytic studies in the literature have been based on the protease resistance of fibrils in the context of amyloidosis [7,35,51,52,53,54]. Recent reports on food fibrils as a potential ingredient in food formulations have shown some extent of resistance of fibrils in whey protein, soy protein, kidney bean isolates, and egg whites, towards proteases such as pepsin, pancreatin, and Proteinase K [55]. Furthermore, comparative studies on crystallin, insulin, and whey protein amyloid fibrils have demonstrated a higher but varying protease resistance of amyloid fibrils towards trypsin, pepsin, and Proteinase K [23]. We assessed the stability of AHB fibrils in the presence of trypsin, and found AHB fibrils to be resistant to trypsin digestion for the first 6 h, with evidence of some fibril digestion after 24 h. The stability of AHB fibrils in the presence of physiologically relevant proteases such as trypsin imparts useful information on how these fibrils can be utilized for potential applications. For some applications, it may be favorable to have a transient scaffold that can be digested by proteases at a fixed time point.

### 3.4. Scaled up Formation of AHB Fibrils from Bovine Waste Blood

Previous scale-up studies on crystallin nanofibers have shown the difficulty in nanofiber formation when the volume was increased up to 40 mL and above. The reduced crystallin nanofiber formation in larger volumes was speculated to be associated with the internal surface area (ISA) of the different containers used in the scale-up process [56]. However, the findings from our scale-up studies show that AHB fibrils could be formed in up to 250 mL volumes, regardless of the reduced surface area.

## 4. Materials and Methods

### 4.1. Materials

Purified hemoglobin was obtained from Sigma (Lot No. 125H9310, Auckland, New Zealand) and bovine waste blood was kindly provided by Taylor Preston Limited (Johnsonville, New Zealand). Unless otherwise specified, all chemicals, reagents, and solvents were purchased from Sigma-Aldrich New Zealand Ltd. (Auckland, New Zealand), BDH Laboratory Supplies (Albany, New Zealand), or Invitrogen (Auckland, New Zealand), and were of analytical grade. All buffers and solutions were prepared with ultra-pure water de-ionized by a Milli-Q (MQ) water system (Millipore, Darmstadt, Germany) and filtered through a 0.45 μm MF membrane (Millipore) under vacuum.

### 4.2. Preparation of Apo-Hemoglobin from Bovine Blood

Hemoglobin was extracted from bovine waste blood by adapting the red blood cell (RBC) lysis protocols employed in the literature [57,58,59]. Bovine blood collected from abattoirs was immediately treated with 10% sodium citrate, followed by centrifugation (3500 rpm for 15 min at 4 °C, Heraeus Multifuge 3S-R centrifuge, DJB Labcare, Buckinghamshire, UK). After centrifugation, the top plasma layer was discarded and the red blood cell (RBC) pellet was incubated with an equal volume of 0.1% sodium chloride for 1 h at 4 °C. Lysed RBCs were centrifuged at 3500 rpm for 30 min at 4 °C. The resulting precipitate was subjected to multiple washes with 0.1% sodium chloride, until a clear supernatant was obtained.

Bovine hemoglobin was processed to form AHB using an acid-butanone phase extraction method [60]. The hemoglobin pellet obtained from RBC lysis was dissolved and diluted in ultra-pure water to obtain an aqueous solution of 10 mg/mL. The solution pH was adjusted to pH 2.8 with 1 M HCl and thoroughly mixed with equal proportions of ice-cold 2-butanone. After phase separation, the butanone layer was discarded and the extraction procedure was repeated until the organic layer was clear. Residual 2-butanone in the aqueous layer was removed by overnight dialysis in ultra-pure water at 4 °C. After dialysis, the AHB solution was concentrated using Amicon Ultra-15 centrifugal filter units (Merck Millipore, Darmstadt, Germany) with a molecular weight cut off (MWCO) of 3 kDa, to obtain a final concentration of 10 mg/mL. The same protocol was followed to obtain a 10 mg/mL AHB solution from commercially available hemoglobin.

### 4.3. LC–MS Analysis of AHB Solutions

The AHB solution prepared from bovine blood was analysed by LC–MS. The pH of the AHB solution was adjusted to 2.0 with 1 M HCl. This sample was analyzed by LC–MS at the Centre for Genomics, Proteomics and Metabolomics at the School of Biological Sciences, The University of Auckland. Samples were first diluted 2500 fold in 0.1% formic acid and 10 μL was injected onto a 0.3 mm Discovery Wide Pore C5 column (Sigma, St. Louis, MO, USA), attached to the ion spray source of a QSTAR XL mass spectrometer (Sciex, Framingham, MA, USA). The following gradient was applied at 6 μL/min: 0–4 min 10%, 24 min 55% B, 27 min 97% B, 30 mins 97% B, 32 min 10% B, and 35 min 10% B, where A was 0.1% formic acid in water and B was 0.1% formic acid in acetonitrile (ACN). A time-of-flight mass spectrometry (TOF–MS) scan was made in a positive ionization mode. Molecular weight profiles were deconvoluted using the Protein Reconstruct Tool within PeakView 2.1 (Sciex).

### 4.4. Fibril Formation from AHB

The salinity of the 10 mg/mL AHB solution was adjusted to 125 mM by the addition of NaCl. The final pH of the solution was adjusted to pH 2.8 with the addition of 1 M HCl and 0.1 M NaOH. The solution was incubated in a volume of 1 mL at 80 °C for 24 h (Thermo Scientific multi-block heater, Waltham, MA, USA). After the incubation period, the samples were allowed to stand at room temperature for 24 h prior to other experiments. The presence of amyloid-specific characteristics in AHB fibrils was assessed by the ThT assay, X-ray fiber diffraction, TEM, and AFM.

### 4.5. ThT Assay

The ThT assay was carried out based on the method of LeVine [61]. ThT dye (2.5 mM) was freshly prepared in buffer containing 100 mM NaCl, 50 mM Tris/HCl, pH 7.5. The prepared dye was filtered and stored under dark conditions for a maximum period of seven days. Fluorescence experiments were carried out in 96-well plates by mixing the buffer, dye, and fibril samples to a total volume of 200 μL, where the final ThT concentration was 25 μM. ThT fluorescence was measured in triplicate for each sample by using an EnSpire Multimode Plate Reader (Perkin Elmer, Waltham, MA, USA) with excitation and emission filters of 450 and 485 nm, respectively [61].

### 4.6. Circular Dichroism

Far-UV CD scans were performed on a Chirascan CD Spectrometer (Applied Biophysics, Troy, NY, USA) at 24 °C using a 1 mm path length quartz cuvette (Hellma Analytics, Müllheim, Germany). Each CD spectrum measurement represents the average of five scans (5 min/scan) obtained with a 1 mm bandwidth. Baseline spectra were collected in water alone, as the samples were prepared in filtered Milli Q water. The baseline values were then subtracted from the raw peptide spectral data. The measurements were performed at a sample concentration of 0.125 mg/mL. CD signals were expressed as molar ellipticity [θ] (deg·cm^2^·dmol^−1^).

### 4.7. AFM

Each AHB fibril sample was buffer exchanged and diluted with ultra-pure water to a concentration of 0.01 mg/mL prior to AFM sample preparation. For AFM imaging, samples were prepared on freshly cleaved mica surfaces by the deposition of 20 μL of diluted fibril solution. After 1 hour, excess solution was pipetted off and the surface was washed with 20 μL of ultra-pure water before leaving to air-dry overnight. AFM was performed using an Asylum Research Cypher ES instrument (Oxford Instruments, Abingdon, UK) and the images were acquired in air using a tapping mode with a Tap150 probe (resonance frequency 175 kHz, force constant 1.5 N/m to 15 N/m) from Budget Sensors (Sofia, Bulgaria). During the post-processing of images, the background of the images was flattened, while the features were preserved by masking. Images were analyzed for height profiles using Gwyddion analysis software [62]. Peak-to-peak distances were calculated from the distances between the fibril height maxima measured from topography traces.

### 4.8. TEM

Fibril solutions were buffer exchanged and diluted 20-fold in ultra-pure water prior to TEM grid preparation. Samples were deposited onto Formvar-coated copper TEM grids (200 mesh) (Electron Microscopy Sciences- Lot No. 150708, Hartfield, PA, USA) and were subsequently stained with 2% uranyl acetate (*w*/*v*) solutions by using the floating grid technique [63]. Grids were analyzed using a FEI Technai 12 electron microscope (FEI company, Hillsboro, OR, USA) operating at 120 kV. Representative images were captured in triplicate using a Gatan ultrascan 1000 camera (Pleasanton, CA, USA) and the processing was carried out in the Gatan digital micrograph software. The mean diameter of the fibrils was calculated by obtaining triplicate diameter measurements of at least five fibrils using ImageJ software [64].

### 4.9. X-ray Fiber Diffraction

For the X-ray fiber diffraction analysis of AHB fibrils, samples were concentrated by centrifuging at 12,500 rpm for 15 min and resuspending multiple pellets in ultra-pure water. The final concentration of the solution was 56 mg/mL. Sample preparation was carried out using methods based on the stretch frame technique with waxed glass capillaries [65]. The fibril stalks obtained were placed in an X-ray beam and diffraction data were collected using a Rigaku007 rotating anode source equipped with an MAR345 image plate detector at the CuKα wavelength of 1.5418 Å (MarXperts, Norderstedt, Germany). The sample to the detector distance was 350 mm and the exposure time was 5 min. Images were analyzed for reflection distances using Adxv software (version 1.9.12, Andrew Arvai, The Scripps Research Institute, La Jolla, CA, USA).

### 4.10. Stability of AHB Fibrils

To test the stability, AHB fibrils were pelleted by centrifugation (15 min at 12,500 rpm) and resuspended in appropriate solvents/buffers to test specific parameters including pH and solvent stability or the appropriate buffer for enzymatic tests. Each stability parameter was assessed at 0, 1, 3, 6, and 24 h time intervals using the ThT assay and TEM imaging. For temperature stability, pre-formed fibrils resuspended in fibrillation buffer were incubated at 80 °C, 37 °C, 22 °C, 4 °C, and −20 °C. In the case of the −20 °C sample, fibril pellets were snap frozen in liquid nitrogen, followed by immediate storage at −20 °C. Samples were brought back to room temperature before being examined by the ThT assay and TEM imaging. The pH stability was evaluated by incubating AHB fibrils in 30 mM glycine (pH 2.0), 30 mM sodium acetate (pH 4.0), 30 mM sodium citrate (pH 6.0), 30 mM Tris HCl (pH 8.0), and 60 mM sodium phosphate (pH 10.0) [7]. In order to assess the solvent stability, fibrils were resuspended in absolute methanol, ethanol, isopropanol, acetonitrile, and dimethylsulfoxide. The impact of proteolytic enzymes on AHB fibril stability was tested in the presence of trypsin. The fibril: enzyme ratio was 20:1 (w:w). Trypsin digestion was carried out in 50 mM Tris/HCl, 1 mM CaCl_2_, pH 8.0, 37 °C. To inhibit the activity of trypsin, ice-cold HCl solution (pH 2.0) was used.

### 4.11. Large Scale Fibril Preparation

For large scale processing, 1 L of blood was acidified to pH 2.8 and allowed to stand in an ice bath for 1 h. The ice-cold solution of acidified blood was mixed with 1 L of 2-butanone in a separating funnel and the mixture was left at room temperature for phase separation. After 30 min, the aqueous layer was separated, followed by a repeated phase extraction with fresh ice-cold 2-butanone. The aqueous layer, containing AHB, was concentrated and separated out of residual 2-butanone with the aid of a rotary evaporator (Rotavapor R110, Buchi, Flawil, Switzerland) at a constant pressure of 37 mBar and a temperature of 40 °C. AHB fibrils were formed under the same conditions used for the 1 mL studies, with the collected aqueous layer diluted to 10 mg/mL. For 250 mL volumes, samples were incubated in a climatic chamber with an MB1 display controller (BINDER, GmbH, Tuttlingen, Germany).

## 5. Conclusions

Driven by their high stability, mechanical properties, and ease of formation, amyloid fibrils have been investigated for numerous applications. Hemoglobin is one of the most abundant proteins in blood and has not been previously shown to form amyloid fibrils. Our studies conclude with sufficient evidence that de-hemed hemoglobin (apo-hemoglobin) forms amyloid fibrils when subjected to pH 2.8, 125 mM NaCl, and 24 h incubation at 80 °C. Stability studies revealed that apo-hemoglobin fibrils can withstand a range of processing conditions, such as extreme temperatures and pHs, organic solvents, and proteases. AHB fibrils were more stable in absolute MeOH, EtOH, MeCH(OH)Me, and MeCN, compared to DMSO, and can be potentially explored for different applications. The stability of fibrils in alcohols can be exploited for microfabrication, whereas the solubility of AHB fibrils in DMSO could be applied in gold nanoparticle alignment, where amyloid fibrils have been used as a biotemplate [66]. The ease of solubility of AHB fibrils in DMSO can replace the need for high-cost techniques such as low pressure “air” plasma for fibril removal [66].

High manufacturing costs associated with amyloid fibrils often restrict further exploitation of these applications at a commercial scale. This paper has addressed the latter issue by utilizing bovine waste blood from the meat industry as a raw material for amyloid fibril formation. Therefore, the combined low manufacturing costs and the stability of apo-hemoglobin fibrils warrant the investigation of these fibrils as functional bionanomaterials for commercial applications. The findings from our studies not only present a solution for minimizing the manufacturing costs of amyloid fibrils by utilizing animal waste blood, but also a logical means of adding value to such a low-cost by-product in the industry.

## Supplementary Materials

The following are available online at www.mdpi.com/2218-273X/7/2/37/s1, Figure S1: SDS-PAGE gel of the different stages in AHB fibril formation, Figure S2: LC–MS analysis of AHB obtained from RBC lysis, Figure S3: ThT fluorescence of amyloid fibrils formed from pure and waste blood AHB, Figure S4: ThT assay and TEM images of hemoglobin amyloid fibrils formed using scaled up protocol.
