# Supplementary material for: Amyloid Fibrils from Hemoglobin
Source: Biomolecules. 2017 Apr 11;7(2):37. doi: 10.3390/biom7020037 (PMC5485726; doi:10.3390/biom7020037)
Supplement: Supplementary file 1 [file biomolecules-07-00037-s001.pdf]

Supplementary information

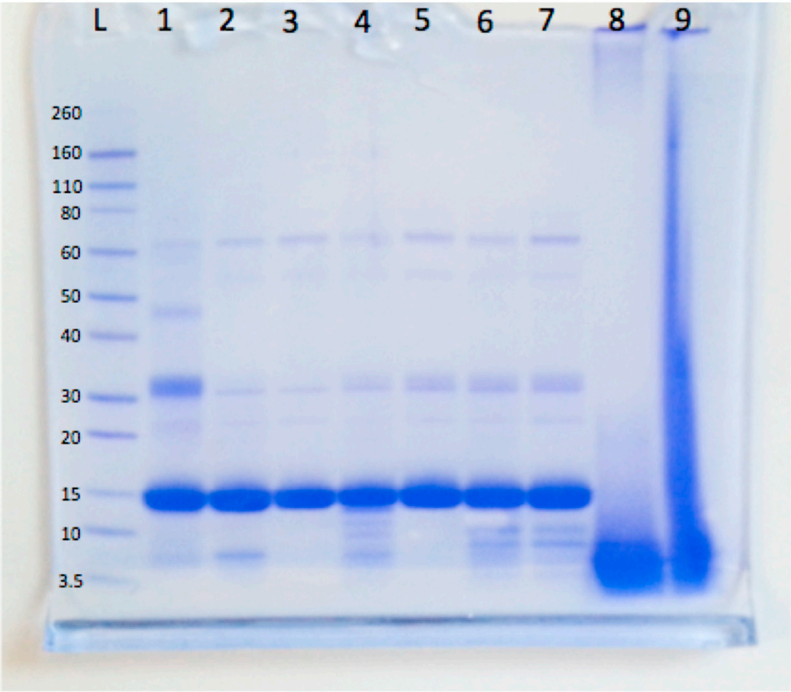

Figure S1. SDS-PAGE gel of the different stages in AHB fibril formation. ***L***= *ladder (molecular weights indicated in kDa on the left-hand side),* ***1***= *sigma hemoglobin (neutral pH),* ***2***= *hemoglobin (whole blood lysis, neutral pH),* ***3***= *hemoglobin (RBC lysis, neutral pH),* ***4***= *AHB at neutral pH (whole blood lysis),* ***5***= *AHB at neutral pH (RBC lysis),* ***6***= *AHB at pH 2.0 (whole blood lysis),* ***7***= *AHB at pH 2.0 (RBC lysis),* ***8***= *AHB fibrils (whole blood lysis),* ***9***= *AHB fibrils (RBC lysis).*

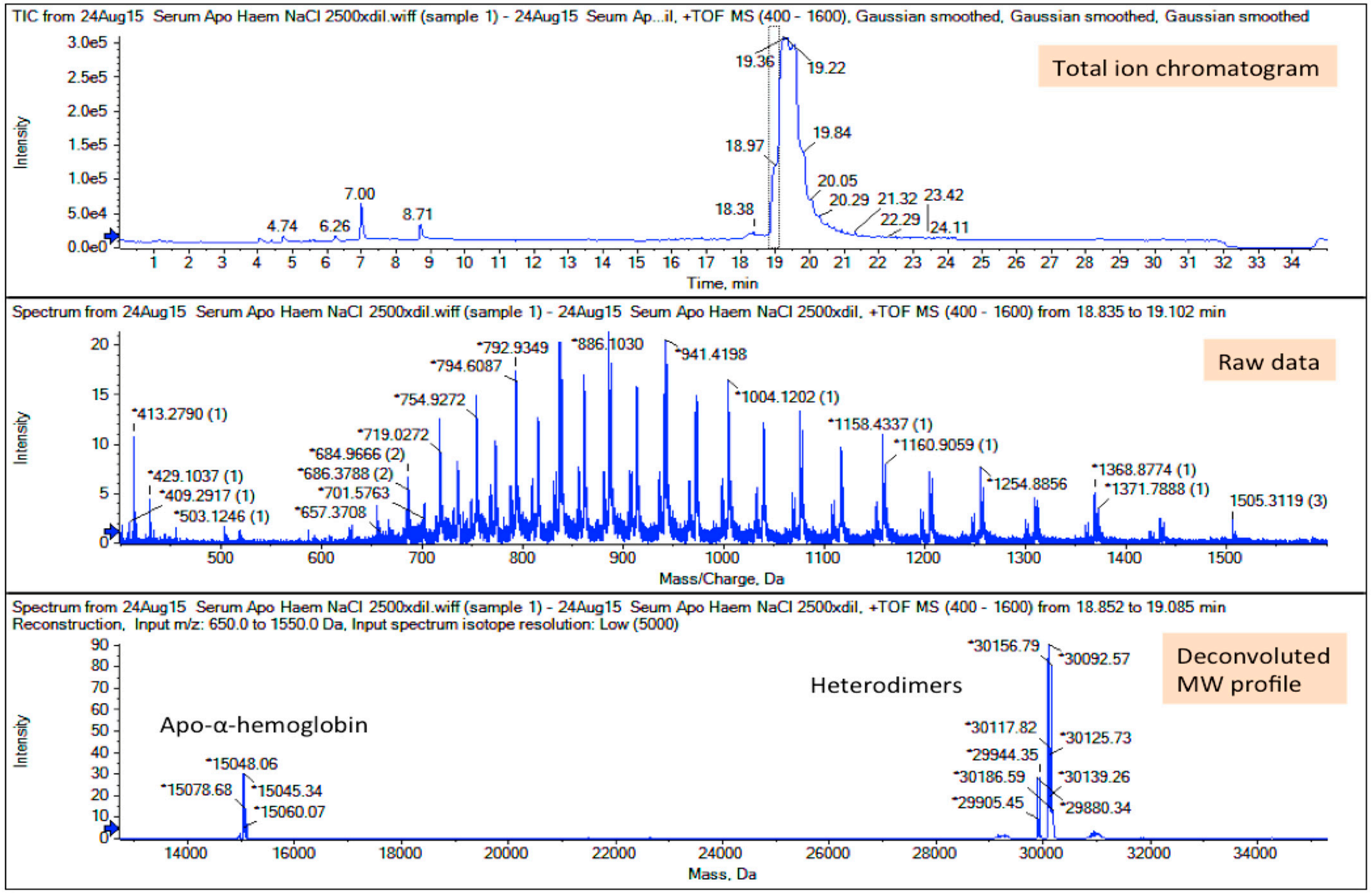

Figure S2. LC-MS analysis of AHB obtained from RBC lysis. Samples were first diluted 2,500 fold in 0.1% formic acid and 10 µL was injected onto a 0.3 mm Discovery Wide Pore C5 column (Sigma, St. Louis, MO, USA), attached to the Ionspray source of a QSTAR XL mass spectrometer (Sciex). The following gradient was applied at 6 µL/min: 0-4 min 10%, 24 min 55% B, 27 min 97% B, 30 mins 97% B, 32 min 10% B, 35 min 10% B, where A was 0.1% formic acid in water and B was 0.1% formic acid in acetonitrile. A TOF-MS scan was made in positive ionization mode. Molecular weight profiles were deconvoluted using the Protein Reconstruct Tool within PeakView 2.1 (Sciex).

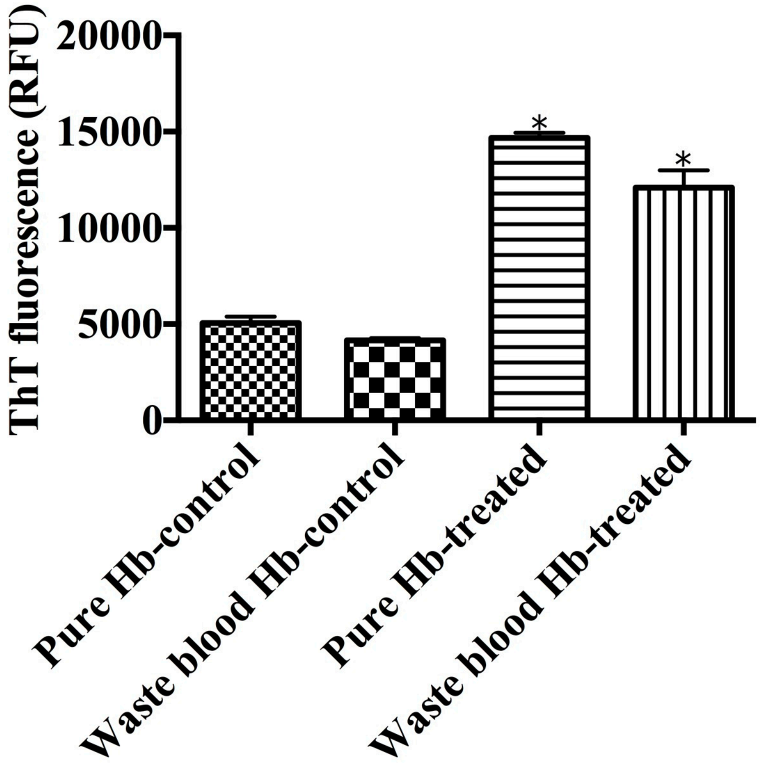

Figure S3. ThT fluorescence of amyloid fibrils formed from pure and waste blood AHB. ThT fluorescence intensity of aqueous AHB solution extracted from pure and waste blood hemoglobin after incubating at pH 2.8, 125 mM NaCl, 80 °C for 24 h. Control AHB samples were incubated at pH 2.8, 125 mM NaCl at room temperature (RT) for 24 h. Each reading represents an average of triplicate well readings, with the error bars representing the standard error of mean. *Significantly different (unpaired T-test) when compared to control, $p<0.05$.

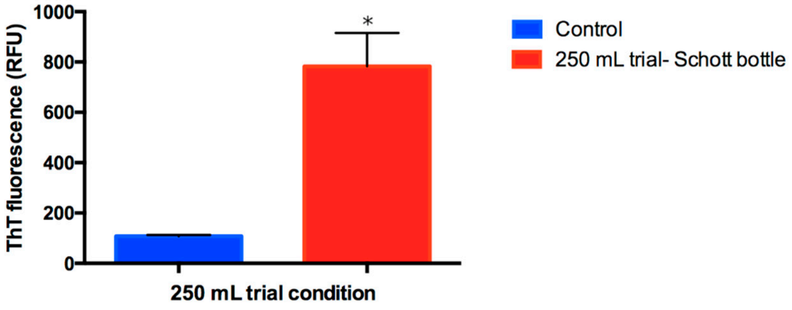

(A)

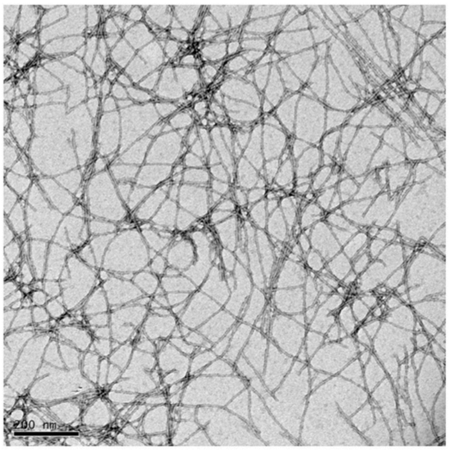

(B)

Figure S4. (A) ThT fluorescence of the 250 mL trial incubated at 80 °C for 24 h at pH 2.8 and in the presence of 125 mM NaCl. ThT readings were reported post-24 h incubation period at room temperature. Each reading represents an average of triplicate well readings of two samples, with the error bars representing standard error of mean. * Significantly different when compared to control, $p<0.05$.

(B) TEM micrograph of 250 mL trial incubated at 80 °C for 24 h at pH 2.8 and in the presence of 125 mM NaCl. The images were captured at 15,000x magnification, post to a 24 h incubation period at room temperature. Scale bars represent 0.2 µm.
